# A long-term localization and mapping system for autonomous inspection robots in large-scale environments using 3D LiDAR sensors

**DOI:** 10.1371/journal.pone.0328169

**Published:** 2025-07-31

**Authors:** Wandeng Mao, Liang Jiang, Shanfeng Liu, Shengzhe Xi, Hua Bao

**Affiliations:** 1 State Grid Henan Electric Power Research Institute, Zhengzhou, Henan, China; 2 State Grid Henan Electric Power Company, Zhengzhou, Henan, China; 3 The School of Artificial Intelligence, Anhui University, Hefei, China; Universidade Federal de Uberlandia, BRAZIL

## Abstract

Inspection mobile robots equipped with 3D LiDAR sensors are now widely used in substations and other critical circumstances. However, the application of traditional LiDAR sensors is restricted in large-scale environments. Prolonged operation poses the risk of sensor degradation, while the presence of dynamic objects disrupts the stability of the constructed map, consequently impacting the accuracy of robot localization. To address these challenges, we propose a 3D LiDAR-based long-term localization and map maintenance system, enabling autonomous deployment and operation of inspection robots. The whole system is composed of three key subsystems: a hierarchical SLAM system, a global localization system, and a map maintenance system. The SLAM subsystem includes Local Map Representation, LiDAR Odometry, Global Map Formulation and Optimization, and Dense Map Generation. Specifically, we construct an efficient map representation that voxelizes only the occupied space and computes local geometry within each voxel. The design of LiDAR Odometry ensures high consistency with this map representation mechanism. Then, to address drift errors, we formulate the global map as a graph of local submaps that undergo global optimization. Furthermore, we utilize marching cubes to generate a mesh model of the map. Our system outperforms the state-of-the-art LiDAR odometry method, LOAM, reducing average absolute position error by 30 % and 38 % on two public datasets. The comparative evaluation highlights the system’s superior accuracy and robustness, and demonstrates its high SLAM ranking in real-world scenarios. For global localization, we propose a novel ScanContext-ICP method, which integrates our improved ScanContext method, termed ScanContext++, for place recognition and global pose initialization. The Iterative Closest Point (ICP) algorithm is then employed for precise point cloud alignment and pose refinement, enabling the recovery of the robot’s position on the offline map when localization is lost. Finally, the map maintenance system tracks environmental changes, distinguishing stable features from dynamic ones. The system assigns higher weight to stable voxels, thereby improving localization accuracy. Furthermore, our time distribution mechanism refines map updates by filtering unstable points through temporal and segment-level analysis, which further enhances map maintenance. We conduct extensive experiments on public datasets to validate our system. The experimental results demonstrate that our system is effective and can be deployed on inspection mobile robots.

## Introduction

For decades, 3D LiDAR sensors have gained tremendous attention in the field of mobile robots due to their stable and wide-ranging environmental perception capabilities [1]. It can assist mobile robot in accurately perceiving the surrounding environment and is less susceptible to interference from external surroundings, making them an integral part of various applications, such as autonomous vehicles, surveillance systems, and most notably, inspection robots. In today’s increasingly automated and intelligent industrial society, inspection robots play an increasingly important role in areas such as facility maintenance, disaster monitoring, and safety inspections. They can perform tasks in environments that are difficult or impossible for humans to access, such as chemical plants, power transmission lines, and underground pipelines. In these applications, 3D LiDAR technology has become an essential component of inspection robot systems due to its ability to provide high-precision distance measurements and environmental perception capabilities under various lighting and weather conditions. 3D LiDAR not only helps robots achieve high-precision positioning and navigation but also provides detailed three-dimensional information about the environment, ensuring the safety and effectiveness of task execution [2].

For inspection mobile robot, it is often designed to operate in large-scale and complex environments, lean heavily on LiDAR sensors to perform long-term autonomous navigation. However, there are still many challenges in the long-term operation of inspection robots. Firstly, managing accumulated errors is a major challenge, especially outdoor or underground environments where external reference points such as GPS signals may be lacking. Ensuring accurate localization over long periods of operation is crucial for practical applications [3]. Secondly, due to the continuous changes and complexity of the environment, robots need to continuously maintain and update their maps during long-duration operations, requiring algorithms with high robustness and flexibility. In addition, the inherent limitations of mobile platforms, including restricted storage, limited computational capability, constrained energy supply, and narrow communication bandwidth, impose additional constraints on long-term operations [4]. As operational time increases, the demand for processing and communication resources grows accordingly, often leading to network delays and data packet loss [5,6]. In general, long-term SLAM must deal with three key issues: maintaining accurate localization over extended periods, performing robust relocalization in dynamic or revisited areas, and handling environmental changes for reliable map maintenance [7,8].

To address the above-mentioned issues, various 3D LiDAR-based localization and mapping systems have been proposed, differing in their approach and scope. Specifically, To realize long-term localization and map maintenance is targeted to include the following modules. Firstly, LiDAR Odometry is an important technique that uses LiDAR scanner data to estimate the motion of a robot. By analyzing the scan data and combining it with prior map information, the robot can perform localization and navigation in unknown environments, avoid obstacles, and reach the target location. For instance, LOAM [9] is a widely adopted LiDAR odometry technique that utilizes feature-based algorithms. It extracts and matches distinctive environmental elements, such as edges or planes, from consecutive LiDAR scans or frames to estimate the robot’s movement. The Suma [10] algorithm adopts the approach of extracting surface element features and utilizes the surface element features of the Surfel map to achieve front-end pose estimation and loop closure detection. The algorithm has been applied in outdoor large-scale scenes and has achieved promising results. Distribution-based approaches, like Normal Distributions Transform (NDT) [11], use algorithms to match the point cloud distributions or geometric properties from consecutive scans without explicitly extracting features. Due to the sparsity of LiDAR points in large-scale scenes, distribution-based odometry methods are generally superior to other methods. Its efficiency can be promoted by parallel computing. However, these methods often require elaborative tuning and additional optimization steps to produce high-accuracy results, which introduces complexity and computational load. To relieve the drift error and improve pose estimation and tracking performance in complex environment, the geometry distribution definition, computation, alignment constraints construction, optimization, and distribution updating mechanism are required to be modified and improved.

Secondly, 3D LiDAR mapping is another crucial mechanism for robot navigation, enabling effective path planning through the construction of maps. This allows for early identification of changes in hazardous road conditions, such as sharp turns [12], to prevent collisions. In terms of mapping, many of the existing 3D LiDAR Simultaneous Localization and Mapping (SLAM) systems, like Google’s Cartographer [13], rely on simple scan aggregation methods to create representations. Such maps, although effective for short-term applications, often prove inadequate for long-term localization, map maintenance, or supporting higher-level tasks. Recently, spatial voxelization and distribution-based representation within each voxel have been widely used for efficient mapping and updating [14,15]. These methods involve dividing the environment into small voxel units and storing environmental information within each voxel. Enabling rapid and efficient map construction and updates, allowing for effective capture of environmental details, and providing accurate navigation information. However, these simplistic approaches often produce sparse or low-resolution maps, which are insufficient for tasks such as fine-grained inspection and obstacle-aware navigation.

Thirdly, technologies for place recognition and map updates struggle to manage environmental changes efficiently. Recently, there exist abundant LiDAR scan descriptors for place recognition, including ScanConext [16], Iris [17], and OverlapNet [18], etc. However, a common problem of these descriptors is that they are less robust to the viewpoint changes. Once a loop is detected, global pose graph optimization is activated to refine the trajectory. This introduces a new challenge: after trajectory correction, the question arises of how to update the voxel-based global map, as the map is heavily dependent on accurate pose estimations. One solution is provided by C-blox [19], which formulates the global map as a graph of local submaps and the global mapping is to maintain the global consistency between submaps.

Finally, with the constructed map, inspection robot can perform global localization, which is essentially more stable than pose tracking in unknown environment. However, due to the environment and its layout change with time, the difference between the global map and the real environment tends to be increased, which introduces risks in the global localization tasks. Thus, the system also takes the responsible for tracking the environment changes and updating the map model immediately. Recent studies have explored different strategies to enhance the robustness of long-term SLAM in such dynamic contexts. For example, Liu *et al*. [20] propose Voxel SLAM, using a unified voxel map structure that supports data association across short-, mid-, and long term sessions and enables consistent multi map alignment through hierarchical optimization. In parallel, Li *et al*. [21] present SD-SLAM, which employs semantics and Kalman filtering to distinguish static, semi static, and dynamic landmarks, significantly improving robustness in urban driving environments.

Despite recent advances, existing systems often focus on a subset of these problems, leading to fragmented solutions that are difficult to generalize or scale. A more unified and adaptable SLAM framework is therefore needed for long-term inspection tasks. Our work aims to bridge these gaps by proposing a more comprehensive system that addresses these challenges from a holistic perspective. In this paper, we propose a fully integrated system for long-term localization and map maintenance. The system comprises three key subsystems: a hierarchical SLAM system, a global localization system, and a map maintenance system. In the hierarchical SLAM system, the map is represented as a geometry-topological graph, where each node corresponds to a submap. Each submap consists of a set of occupied voxels, with Signed Distance Field (SDF) values computed for each voxel. These voxels are efficiently stored and retrieved using a hash table. Our LiDAR odometry is adapted to accommodate this novel map representation. The generation of a dense map is performed in two stages: first, the submap is integrated into a global SDF map, followed by VDB-Fusion to obtain a mesh model of the global environment. The global localization system is an extended version of our LiDAR odometry and the global pose initialization is based on our proposed ScanContext-based Iterative Closest Point (ICP). This novel ScanContext-ICP method not only improves robustness under significant viewpoint changes but also enhances localization accuracy, particularly in large-scale environments. The map maintenance system is achieved by updating the geometry and observation information in each voxel. Besides, the system controls the voxel life according to its creation timestamp, observed times, last observed timestamp and difference with the map time. Only the healthy voxels are used for localization and map processing. The main contributions of this work are the following:

- A hierarchical 3D LiDAR SLAM system is proposed that can be applied in large-scale environments, effectively reducing drift errors and enabling fast map updates.
- A Long-term map maintenance mechanisms are proposed, which can keep tracking the environment changes and thus benefit the global localization performance. During the global localization and initialization process, we propose a novel ICP based on ScanContext++ that can be used for global point cloud registration and reduce the cost of the scan matching process.
- A dense mapping model is incorporate into our system, enabling us to construct a high-quality mesh model of the global map.

This paper is organized as follows: a review of related work is provided first. Then, we introduce our approach and methodology. The experimental evaluation is subsequently presented to validate the proposed system. Finally, we summarize the main findings and outline future research directions.

## Related work

The existing SLAM systems can be classified into three classes according to their map representation, including feature-based methods, voxel-based methods and local map-based methods.

### Feature-based method

Feature-based methods [10,22–28] construct maps by identifying and extracting prominent feature points or feature descriptors from the environment. Different from some methods that utilize visual SIFT for detecting environmental features [29], LiDAR-based SLAM approaches obtain edge and plane feature points by calculating the curvature of each point. LOAM [9] is a widely recognized and classic LiDAR SLAM algorithm. It incorporates motion distortion correction and scene structure recognition using the abundant information available in high-resolution LiDAR data, creating a real-time system. LOAM employs parallel processing of features during mapping and localization, guaranteeing real-time performance and accuracy. To further improve the efficiency of ground vehicle SLAM, some researchers have made improvements based on LOAM. Lego-LOAM [30] optimizes specific features by subdividing the received point cloud data and reconstructing complex terrain. This enables Lego-LOAM to perform well in ground vehicle applications where high-density point clouds are not required. LIO-SAM [31] builds upon Lego-LOAM by providing a factor graph-based optimization framework that addresses the alignment issue between high-frequency IMU and low-frequency LiDAR data. This gives LIO-SAM strong information fusion capabilities, allowing it to achieve good trajectory estimation in noisy and challenging environments. MULLS [32] utilizes multiple LiDAR setups to achieve high-precision and robust localization and mapping capabilities, particularly in complex and dynamic indoor-outdoor environments. The core idea behind MULLS is that LiDAR data from multiple viewpoints and distance scales can complement each other, helping to reduce coverage blind spots and data sparsity issues that a single LiDAR device may encounter. F-LOAM [33] algorithm employs a two-step approach to correct distortion in raw point cloud data. During the nonlinear optimization process to solve for pose estimation, it applies weighted constraints on feature points, deviating from the parallel processing of LiDAR odometry and mapping threads in the LOAM algorithm. The BALM [34] algorithm introduces the Bundle Adjustment (BA) framework into LiDAR SLAM. It establishes a BA optimization model on sparse LiDAR feature points, including edges and planes. By directly minimizing the distances between feature points and edges/planes, the BA optimization is eventually reduced to handling the pose of a single LiDAR frame. This method utilizes feature points to construct maps, which increases computational efficiency. However, the limited nature of sparse map information prevents it from providing detailed obstacle and other relevant information required for supporting complex path planning. As a result, sparse maps are not suitable for the subsequent navigation tasks of inspection robots.

### Voxel-based method

The map of above approaches is represented as a set of geometry feature points and the LiDAR odometry is achieved by minimizing the feature-to-feature matching distance. The global map is updated by directly aggregating the feature points, which tend to become redundant in long-term mapping process and thus burden the mapping efficiency. To alleviate such problem, voxel-based SLAM systems [35–43] formulate the map by occupied voxels. In the voxel, various information is encoded, such as line and plane features, surfels, approximated Gaussian distributions, etc. Ref. [44] utilizes a combination of LiDAR and a 3D voxel map constructed from outdoor point clouds for mobile robot localization. By integrating odometry and the 3D voxel map, the complete 6D state of the mobile robot can be estimated, achieving a high level of accuracy. Ref. [45] presents a voxel-based localization and mapping system that achieves real-time trajectory estimation for multi-robot systems in GPS-denied environments. This system formulates the registration problem as a Maximum A Posteriori (MAP) problem and simplifies it into a nonlinear least squares problem using Gaussian approximation and Principal Component Analysis (PCA). By utilizing a voxel-based matching approach, the computational efficiency is significantly improved, and the communication bandwidth of the localization and mapping system is reduced. Ref. [46] proposes an adaptive voxel construction method that can adapt to structurally diverse environments. This method utilizes an octree hash data structure to store the voxel representation, which improves the efficiency of voxel construction, updates, and queries. By adapting the size of the voxels to the changing environment, this method achieves a more flexible and efficient representation of the environment’s structure. VoxelMap++ [47] proposes a voxel mapping method with planar merging, where the map is represented using voxels. It utilizes a merging module to distinguish coplanar relationships within various voxels. This approach effectively improves the accuracy and efficiency of LiDAR SLAM. Sparse space voxelization improves the map scalability and significantly reduce the redundancy. Dense mapping methods rely on full-space voxelization. They compute the SDF values of the voxels passed through by LiDAR beams. Compared to sparse mapping methods, it can provide more detailed and accurate map information. But it also require more computational resources, which can affect the real-time performance of robot navigation tasks. Meanwhile, A common problem of these methods is that such mapping framework cannot well process drift errors. If the global trajectory is corrected by loop closure, all the associated voxels should be recomputed, which requires huge computational cost.

### Local map-based method

Recently, local map-based mechanisms have been proposed. The global map is regarded as a set of local maps and the system focuses on maintaining the local maps and only fuse them into the global map when necessary. Karto SLAM [48] converts the data scans collected during the robot’s movement into submaps, with each submap corresponding to the robot’s observation data at a specific moment. When the robot moves to a new area, a new submap is created. The accuracy is improved by optimizing these local submaps. RatSLAM [49] divides the environment into many smaller regions or modules, each with its own local map. These local maps are linked together through relative pose relationships. Their main advantages are their robustness and ability to operate internally in large-scale environments, enabling precise long-term localization and mapping even in dynamically changing environments. A method for adaptive local map maintenance has been proposed [50], allowing the system to autonomously prune redundant local maps and render a global occupancy map from multiple local maps. This ensures the robustness and stability required for long-term mapping. Rtabmap [51] creates multiple local map systems, and when a loop closure occurs, it reassembles the newly optimized pose into the global map based on all the local maps in the map set. Additionally, in order to avoid restarting the mapping process from scratch when the robot loses localization, multi-session mapping allows for initializing a new map first, and if a previously visited location is encountered, the two maps are merged. This approach ensures that the mapping and localization process can be seamlessly continued even after temporary localization failure. Such mechanisms significantly improve mapping scalability and address the challenge of high computational cost in global optimization by efficiently utilizing limited computing resources. However, the mechanism should be sophisticated designed to be adapted to the real environment.

Long-term 3D map management is another fundamental capability required by a robot to reliably navigate in the non-stationary real-world. LT-mapper [52] develops open-source, modular, and readily available LiDAR-based lifelong mapping for urban sites. This is achieved by dividing the problem into successive subproblems: multi-session SLAM (MSS), high/low dynamic change detection, and positive/negative change management. Other dynamic removal methods are also proposed. These methods can be classified into the follows categories, ray-casting-based [53,54], visibility-based [55,56], and change-detection-based methods [57,58]. All these strategies strive to provide a perfect high-dynamic and low-dynamic point definition and aim to distinguish them according to specific rules. However, due the LiDAR points sparsity and only range information being encoded, all their experimental results demonstrate that there does not exist an unify geometry rule set that can fully avoid misclassification. Furthermore, most of these methods perform dynamic removal at voxel and point level, which neglects the integration of the object. Thus, in this paper, we propose novel mechanism aiming to distinguish unstable points at time dimension and segment level. The key motivation behind our approach is as follows: for highly dynamic objects, observations exhibit inconsistencies in both spatial and temporal dimensions, while for low-dynamic objects, inconsistencies primarily occur over time. Therefore, we leverage temporal cues to effectively distinguish between them.

The biggest difference between our method and other SLAM methods lies in the fact that traditional SLAM methods often encounter challenges in large-scale dynamic inspection environments due to the interference from dynamic surroundings and potential limitations in LiDAR accuracy. To address these issues, we have drawn inspiration from Voxel-based methods. By extracting environmental information and incorporating algorithms for dynamic object detection and removal, we effectively minimize the interference caused by moving objects. Additionally, we utilize a local map-based approach to enhance the efficiency and robustness of point cloud registration, enabling accurate long-term robot localization and map management.

## Proposed system

### System framework

**Fig 1:**
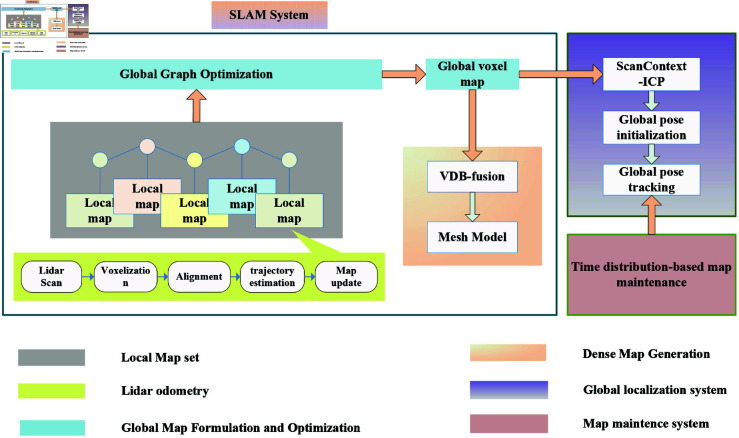
System framework.

The framework of our system is presented in Fig 1, which includes three subsystems, hierarchical SLAM system, global localization system, and map maintenance system. The aim is to achieve long-term self-localization and map maintenance for inspection equipped with 3D LiDAR sensor. The SLAM subsystem consists of the following modules: Local Map Set, LiDAR Odometry, Global Map Formulation and Optimization and Dense Map Generation. Its primary function is to construct both the raw map and dense map of the task environment. Additionally, by leveraging the use of submaps and graph optimization, it effectively mitigates drift errors in large-scale environments. The global localization system is responsible for relocating the robot on the map during the inspection process and performing global pose initialization when the robot is kidnapped or there is a sudden change in the environment. The map maintenance system takes responsible for tracking the environment changes and distinguish stable background, which benefits the global localization performance.

### Hierarchical SLAM system

#### Local map representation.

In our system, local map is used to model local scene centered at an anchor point $[a_{x},a_{y},a_{z}{]}^{T}$ within a certain distance. This local map plays a vital role in the SLAM system by providing a compact, voxel-based, and up-to-date geometric representation of the environment surrounding the robot. It facilitates efficient point cloud registration and supports real-time pose tracking. Compared to global representations, the local map maintains only a limited portion of the environment around the current pose, which significantly reduces memory usage and computational load while preserving sufficient geometric detail for accurate localization. This design is particularly well-suited for long-term deployment or large-scale environments, where frequent global map updates are computationally expensive and difficult to maintain.

Specifically, the local map stores LiDAR points in a voxelized structure centered at the anchor point. Given a set of LiDAR scans that have been transformed into the global coordinates, we assign them into a local map ${ℳ}_{l}^{k}$. Let ${𝒫}_{t}=\{{𝐩}_{i},i=1,\cdots ,N\}$ denote one LiDAR scan. For each point ${𝐩}_{i}=[x_{i},y_{i},z_{i}{]}^{T}$, we compute its corresponding voxel index $I_{v}$ using Eq (1).

$$I_{x}=(x_{i}-a_{x})/g;I_{y}=(y_{i}-a_{y})/g;I_{z}=(z_{i}-a_{z})/g;I_{v}=hash(I_{x},I_{y},I_{z});$$ (1)

where hash(·) is a hash function and *g* is the voxel size. Only occupied voxels are stored in the hash table, significantly reducing memory consumption. Importantly, we construct the hash table within the local map rather than the global map, which effectively mitigates hash collisions and ensures spatial locality for fast lookup during point cloud registration. After all the points are assigned into the voxels, one voxel can be represented using Eq (2).

$$V_{m}=\{{𝐜}_{m},{𝐧}_{m}\}.$$ (2)

where **c** is the center point and **n** is the normal of the contained points in $V_{m}$. This representation preserves local geometric structure, which is essential for reliable point cloud alignment in subsequent ICP refinement.

#### LiDAR odometry.

Given a new LiDAR scan ${𝒫}_{t}$, LiDAR odometry is to optimize its global pose. Firstly, we transform the scan in the global coordinates using an initial global pose guess, which is generally obtained by constant velocity model. Then, for each point ${𝐩}_{i}\in {𝒫}_{t}$, we compute its corresponding voxel $V_{m}$ and access to the voxel by hash table. Finally, we compute the point-to-surface-based constraints using Implicit Moving Least Squares (IMLS)-based mechanism. Specifically, we compute the point-to-surface distance using Eq (3).

$$D_{i}=\frac{\sum_{V_{j}\in 𝒩(V_{m})}W_{j}({𝐩}_{i})\cdot ({𝐩}_{i}-{𝐜}_{j}{)}^{T}\cdot {𝐧}_{j}}{\sum_{V_{j}\in 𝒩(V_{m})}W_{j}({𝐩}_{i})},$$ (3)

where $𝒩(V_{m})$ denotes the neighboring voxels of $V_{m}$ and $W_{j}({𝐩}_{i})$ is defined as Eq (4).

$$W_{j}({𝐩}_{i})=\exp(\frac{\|{𝐩}_{i}-{𝐜}_{j}{\|}^{2}}{2h^{2}}),$$ (4)

where *h* is a cofficient. The weight is decreased with the distance to ${𝐩}_{i}$. Then, the ICP cost function is constructed as Eq (5).

$$\sum_{p_{i}\in {𝒫}_{t}}\|{𝐧}_{m}^{T}\cdot (𝐑{𝐩}_{i}+𝐭-{\hat{𝐩}}_{i}){\|}^{2},$$ (5)

where **R** and **t** are the two pose transformation parts and ${\hat{𝐩}}_{i}$ is defined as Eq (6).

$${\hat{𝐩}}_{i}={𝐩}_{i}-D_{i}\cdot {𝐧}_{m}.$$ (6)

${\hat{𝐩}}_{i}$ is defined as the estimated corresponding point of ${𝐩}_{i}$ on the surface. ${𝐧}_{m}$ is the surface normal estimation at ${\hat{𝐩}}_{i}$. Then, Gauss-Newton method is implemented to compute the optimal (𝐑,𝐭) of Eq (5). In this paper, the IMLS-based constraints are computed by voxel-wise instead of point-wise strategy, which avoid computing point-wise normal estimation. Normal in each voxel can be computed more accurately and voxel-wise strategy makes the cost function construction more efficient. After the current LiDAR scan are aligned with the local map by the proposed LiDAR odometry, the LiDAR points are then used to update the center point and normal of their corresponding voxels. For voxel $V_{m}$, we define its stability as Eq (7).

$$s_{m}=dot(n_{m}^{t-1},n_{m}^{t}),$$ (7)

where dot(·) returns dot product of two normalized normals. If $n_{m}^{t-1}$ and $n_{m}^{t}$ closed, it means that adding new observations to the voxel does not change the normal too much and the voxel is defined as stable voxel. Thus, we also incorporate the index into the cost function as Eq (8). The pseudo code of the LiDAR odometry algorithm as shown in Algorithm 1.

$$\sum_{p_{i}\in {𝒫}_{t}}s_{m}\cdot \|{𝐧}_{m}^{T}\cdot (𝐑{𝐩}_{i}+𝐭-{\hat{𝐩}}_{i}){\|}^{2}.$$ (8)

**Algorithm 1** LiDAR Odometry Algorithm

**Input:** A LiDAR scan ${𝒫}_{t}$

**Output:** Optimized global pose *X*_*i*_ and Updated map *M*_*i*_

1: initial pose *X*_0_ and transform the scan to global coordinates

2: **for** each LiDAR point ${𝐩}_{i}\in {𝒫}_{t}$ **do**

3:   Find its corresponding voxel $V_{m}$ using a hash table

4:   Compute its voxels weight $W_{j}({𝐩}_{i})=\exp\left(\frac{\|{𝐩}_{i}-{𝐜}_{j}{\|}^{2}}{2h^{2}}\right)$

5:   Compute point-to-surface distance

$D_{i}=\frac{\sum_{V_{j}\in 𝒩(V_{m})}W_{j}({𝐩}_{i})\cdot ({𝐩}_{i}-{𝐜}_{j}{)}^{T}\cdot {𝐧}_{j}}{\sum_{V_{j}\in 𝒩(V_{m})}W_{j}({𝐩}_{i})}$

6: **end for**

7: Minimize distance residuals σ using implicit moving least

squares((IMLS)) for point cloud registration

8: Construct ICP cost function $\sum_{p_{i}\in {𝒫}_{t}}\|{𝐧}_{m}^{T}\cdot (𝐑{𝐩}_{i}+𝐭-{\hat{𝐩}}_{i}){\|}^{2}$

9: Compute optimal Pose Transformation Matrix (𝐑,𝐭) using

Gauss-Newton method

10: Align current LiDAR scan with local map using the optimized

pose $X_{i}=X_{0}\cdot (R,t)$

11: Compute voxel stability $s_{m}=\text{dot}(n_{m}^{t-1},n_{m}^{t})$

12: Incorporate stability into the cost function

$\sum_{p_{i}\in {𝒫}_{t}}s_{m}\cdot \|{𝐧}_{m}^{T}\cdot (𝐑{𝐩}_{i}+𝐭-{\hat{𝐩}}_{i}){\|}^{2}$

13: Use LiDAR point cloud to update the center point *c* and

normal vector *n* of each corresponding voxels $V_{m}$.

14: Update the voxel map *M*_*i*_.

15: end.

#### Global map formulation and optimization.

Our global map is formulated as a set of local maps. Specifically, we denote a local map as Eq (9).

$${ℳ}_{k}=\{{𝒯}_{k},{ℬ}_{k},{𝒱}_{k}\},$$ (9)

where ${𝒯}_{k}$ is the global pose transformation of the anchor coordinates of the local map. In each local map, the anchor coordinates are regarded as reference coordinates. ${ℬ}_{k}$ represents the space region, which is generally defined as a 3D bounding box centered at the anchor point. ${𝒱}_{k}$ is the set containing all the occupied voxels and is organized by std::unordered_map. The global map is then represented as Eq (10).

$${ℳ}^{g}=\{{ℳ}_{k},\{{𝐓}_{k_{0}}^{k_{1}}\},k=1,\cdots ,K\},$$ (10)

where ${𝐓}_{k_{0}}^{k_{1}}$ represents the relative pose transformation between the anchor poses of the *k*_0_-th and *k*_1_-th local map. During the mapping process, if the current LiDAR observation exceeds the range of the current local map, then a new local map is initialized and activated. Generally, the anchor pose is set as the current LiDAR pose. With such mechanism, there exists overlap between local maps. For LiDAR points belonging to multiple local maps, we update the corresponding voxels in each local map separately. In this paper, we use ScanContext for loop detection, which is achieved by computing the ScanContext distance between the current scan and the history keyframes. If the distance is smaller than a threshold and keyframe timestamp is not close to the current time, loop is detected and loop closure is performed. Specifically, we firstly construct anchor pose graph and perform global graph optimization [59]. Then, based on the optimized results, we perform locally trajectory estimation within each local map. The local constraints is the relative pose transformation between the local maps. After the hierarchical map optimization, the drift errors can be significantly reduced and the global voxel map can be generated by merging all the local maps. Essentially, the map merging process is to fuse the voxel information located in the overlap areas between neighboring local maps. Benefitted from the hash table, it is efficient to retrieve the overlapped voxels.

#### Dense map generation.

To generate dense map is highly required in inspection tasks. There exist lots of methods for dense mapping in large-scale environment. However, their mapping process is achieved by incrementally integrating LiDAR observations. In this work, we directly derive dense map from our global voxel-based map. Firstly, since each voxel has normal and points’ center, we can easily compute the voxel center’s signed distance Function (SDF). The SDF value of a voxel center point **p** can be determined using Eq (11).

$$SDF(p)=\min_{q\in \partial \Omega }\|p-q{\|}_{2}\cdot sgn(p)$$ (11)

where *p* represents the position of the voxel center point, Ω represents the entire three-dimensional shape, ∂Ω is the surface (boundary) of the shape, *q* denotes the position of any point on the shape’s surface, and sgn(·) is a sign function which reflects the position of point *p* relative to the shape Ω. Then, we incorporate VDBFusion [60] as a module in our system and all the voxels in our map are directly mapped to the voxel map in VDBFusion. In contrast to most state-of-the-art SLAM and mapping approaches, VDBFusion makes no assumptions on the size of the environment nor the employed range sensor. Unlike most other approaches, VDBFusion introduces an effective system that works in multiple domains using different sensors. They build upon the Academy-Award-winning OpenVDB library used in filmmaking to realize an effective 3D map representation. Based on this, their proposed system is flexible and highly effective and, in the end, capable of integrating point clouds and generate dense model of the environment.

### Global localization system

After constructing the global map, if the inspection robot encounters human interference or sudden environmental changes that cause it to lose its position, it becomes necessary to re-localize the robot within the constructed global map to restore its localization. In this section, we focus on introducing our ScanContext-ICP, which integrates ScanContext++ into the ICP framework for global point cloud alignment and localization. This process consists of two key steps: global pose initialization and pose tracking. Firstly, the robot needs to obtain an accurate initial pose transformation based on the previously constructed offline map and the current LiDAR scan data. This initial estimation is crucial for accurately localizing the robot within the prior map.

To improve the accuracy of the initial pose estimation, we propose a modified version of ScanContext, termed as ScanContext++. In ScanContext, the LiDAR points are transformed into a matrix and each element corresponds to 2D square ground block. Each element is defined as the maximum z-value of the points mapped to the corresponding block. However, this approach leads to a significant loss of geometric information. In ScanContext++, each element stores an approximated Gaussian distribution of the contained points, represented by the mean and standard deviation, as defined in Eq (12).

$$e_{\left(i,j\right)}=(\mu_{\left(i,j\right)},\sigma_{\left(i,j\right)}^{2})$$ (12)

where $\mu_{\left(i,j\right)}$ is the mean of the z-values of the points mapped to the block, and $\sigma_{\left(i,j\right)}^{2}$ is the square of the standard deviation of the z-values of these points.

For a LiDAR scan, we evenly sample points on the ground and construct local ScanContext++ descriptor centered at each sampled point. To match two LiDAR scans, we construct their sampled points correspondences by computing their local ScanContext++ distance. The distance between the corresponding elements *e*_(*i*,*j*)_ and $e_{\left(i,j\right)}^{\prime }$ is computed using the Gaussian distribution distance, as shown in Eq (13).

$$d_{B}(e_{\left(i,j\right)},e_{\left(i,j\right)}^{\prime })=\sqrt{1-\frac{1}{\sqrt{\sigma_{\left(i,j\right)}^{2}\sigma_{\left(i,j\right)}^{\prime 2}\cdot 2\pi }}\cdot \exp\left(-\frac{1}{4}\cdot \frac{(\mu_{\left(i,j\right)}-\mu_{\left(i,j\right)}^{\prime }{)}^{2}}{\sigma_{\left(i,j\right)}^{2}+\sigma_{\left(i,j\right)}^{\prime 2}}\right)}$$ (13)

Then, Singular Value Decomposition (SVD) and fine-ICP are performed to obtain the pose transformation given the point correspondence.

Secondly, once the initial pose is initialized, it is essential to continuously track the robot’s pose changes to ensure that its position is updated in real-time with the changing environment. We adopt the Scan-to-Map approach, where the LiDAR’s point cloud data is iteratively matched to the offline point cloud map near the initial pose using ICP. Since we have constructed LiDAR odometry before, the pose tracking can directly leverage odometry data for global localization. In the event of tracking failure (e.g., ICP convergence failure), we will repeat the initial localization process to re-localize the robot, using the ScanContext++ descriptor to recover the robot’s initial pose.

### Map maintenance system

**Fig 2:**
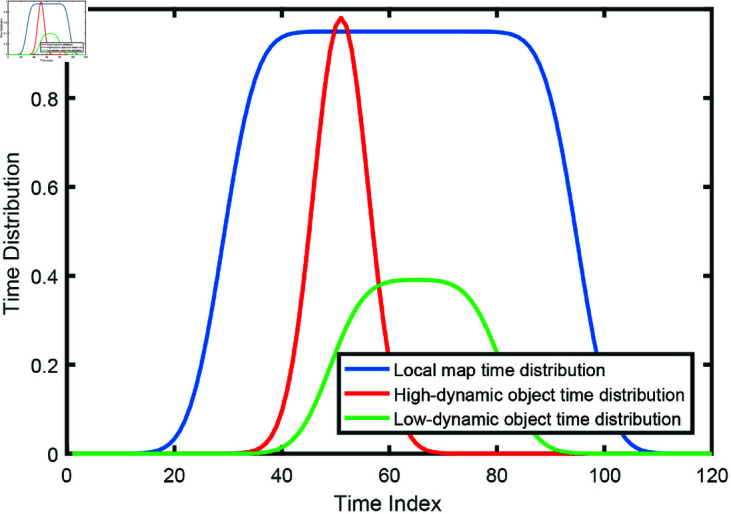
Illustration of the time distribution.

The task environment is dynamic and it is important to make the global map keep tracking of the environment. Though the environment changes can be caused by many reasons, these changes can be generally described as follows. Some occupied voxels turned into free voxels due to object moving or free voxels turned into occupied voxels due to new objects added. It is critical to distinguish these changes of voxels and accurately update their state. A common mechanism is to track all the traversed voxels using ray-casting algorithm. However, it is time-consuming. In this paper, we propose a time distribution-based map maintenance mechanism. Specifically, instead of processing each voxel, we use a prior knowledge that the changes often happen at object level. Thus, we perform LiDAR segmentation in each local map, and associate voxels with each segment. Then, for each segment, the observed timestamps are stored in a set 𝕋. These timestamps are discrete values. Then, we define time distribution ${𝒟}_{s}(T)$ as shown in Eq (14).

$${𝒟}_{s}(T)=\sum_{t_{i}\in 𝕋}𝒩(t_{i},\sigma_{i}),$$ (14)

where 𝒩 denotes Gaussian distribution. It means that the time distribution of one segment is regarded as a mixture Gaussian distributions and each Gaussian distribution is defined by each observed timestamp. $\sigma_{i}$ is the hyperparameter. Then, the time distribution ${𝒟}_{m}(T)$ of the local map can also be easily constructed by counting the contained LiDAR poses’ timestamps following the same strategy. Then, we compute the Wasserstein Distance between time distribution of each segment and the local map. If the distance is larger than a threshold, it indicates that the corresponding segment is dynamic. For instance, as shown in Fig 2, the time distribution segments of high-dynamic and low-dynamic objects are considered dynamic segments. The dynamic objects within these segments will be removed from the map.

Essentially, such mechanism aims to extract segments that are relatively frequently observed through time distribution comparison. For a dynamic object, its timestamps are distributed in peak, while the local map’s time distribution is flat in the time space. For a static object that is removed later, its time distribution is missing on the near time space. Thus, through time distribution distance computing, we can distinguish the state of the segment, and thus the corresponding voxels. Actually, performing map maintenance at the segment level can effectively avoid voxel misclassification, since the voxel state judgement is more prone to errors.

## Experiment evaluation

### Experiment setup

To validate the proposed system and mechanisms, we conducted experiments using public datasets, including KITTI [61] and M2DGR [63]. The environmental conditions during testing included a variety of outdoor settings such as urban streets, highways, and rural areas. These sequences were recorded in daylight, with ambient lighting conditions varying based on weather and time of day. In some sequences, light rain and varying sunlight conditions were present, which may have influenced the quality of the point cloud data. All experiments were performed using the Velodyne-VLP32 LiDAR configuration, with a scanning frequency of 10 Hz, horizontal FOV of 360°, and a vertical FOV of ±30°. The LiDAR sensor had a range from 0.1 m to 100 m, and data was collected at a resolution of 0.5° per scan. The hardware setup consisted of an Intel i5-10400F 4.30 GHz processor with 16 GB RAM, which provided sufficient computational power to process the LiDAR data and run the localization algorithms concurrently. The software components, running on Robot Operating System (ROS) Noetic on Ubuntu 20.04, interacted with the hardware to process the raw LiDAR data in real time. The LiDAR driver read the point cloud data from the sensor and sent it to the software system for processing, where the proposed algorithms, including LiDAR Odometry and ScanContext-ICP, were applied for localization and mapping. Real-time data processing was essential for the system’s performance. To achieve this, the software was optimized for efficiency by employing asynchronous message passing between ROS nodes, enabling prompt handling of incoming sensor data while minimizing latency and ensuring the synchronization of asynchronous information. Data processing and map generation were carried out using the PCL point cloud library(version 1.11.0). Qualitative and quantitative evaluations were performed, and since our system is a combination of multiple subsystems, we evaluated each subsystem individually to assess its contribution to the overall performance.

### Trajectory estimation of our SLAM system

The KITTI datasets are widely used public datasets for research in autonomous driving and SLAM. They provide a large amount of sensor data, including LiDAR, cameras, inertial measurement units (IMUs), GPS, and high-precision ground truth annotations. The datasets cover various large-scale scenarios such as urban streets, rural roads, and highways, enabling researchers to design and evaluate trajectory generation algorithms. We compared our proposed method with LiDAR Odometry and Mapping (LOAM) [9], Fast LiDAR Odometry and Mapping (F-LOAM) [33], and Bundle Adjustment for LiDAR Mapping (BALM) [34], which are well-established LiDAR SLAM algorithms, on nine different sequences from the KITTI dataset. The average error (mean), median error, and root mean square error (RMSE) are shown in Table 1, evaluated using absolute trajectory error (ATE) [62]. The BALM algorithm performed poorly on some sequences and failed, likely due to the high-speed vehicle motion during data collection, which caused a loss of feature points and led to the algorithm’s failure. In contrast, our method demonstrated higher accuracy compared to the other three algorithms.

**Table 1:** Error comparison in the KITTI datasets (in units of meters).

Sequence	LOAM			F-LOAM			BALM			OURS		
	MEAN	MEDIAN	RMSE	MEAN	MEDIAN	RMSE	MEAN	MEDIAN	RMSE	MEAN	MEDIAN	RMSE
Kitti_00	6.877	5.414	8.164	6.992	5.376	8.367	19.907	15.414	24.111	4.442	3.549	5.096
Kitti_01	21.547	22.164	24.210	20.719	21.269	23.345	×	×	×	19.156	20.094	21.063
Kitti_02	74.589	71.131	81.675	16.479	14.408	19.242	×	×	×	**6.881**	**5.713**	**7.770**
Kitti_04	0.211	0.184	0.239	0.186	0.168	0.205	0.798	0.785	0.927	0.247	0.223	0.284
Kitti_05	3.135	2.419	3.649	3.359	2.868	3.850	10.666	9.620	12.074	2.042	1.748	2.255
Kitti_07	0.607	0.537	0.669	0.508	0.457	0.565	1.423	1.074	1.605	0.801	0.732	0.920
Kitti_08	6.982	6.934	7.387	6.997	6.955	7.398	×	×	×	3.831	3.925	3.994
Kitti_09	12.993	12.342	14.708	12.663	11.899	14.379	17.681	17.497	19.746	7.782	5.297	9.580
Kitti_10	3.757	3.072	4.466	3.733	3.030	4.438	×	×	×	1.833	1.586	2.110

**Fig 3:**
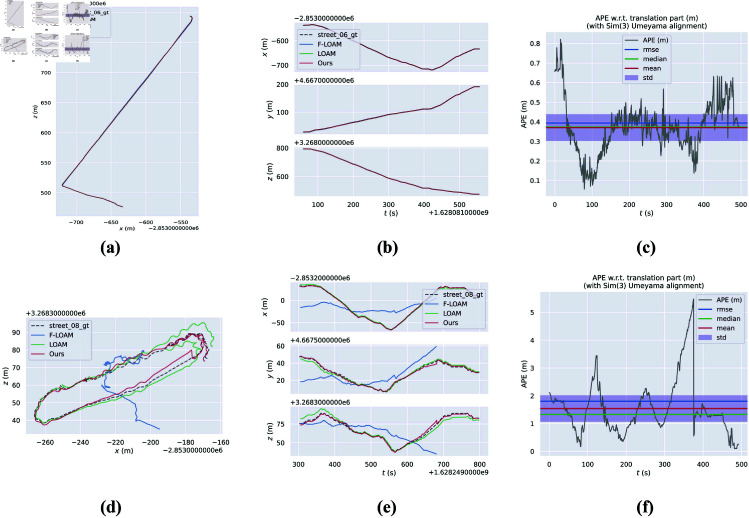
The experimental results on M2DGR dataset. Our method displays a solid red line trajectory, with the ground truth as a black dashed line, LOAM’s estimate as a solid green line, and F-LOAM’s trajectory represented by a solid blue line for comparison. (a) The trajectory of the street06 sequence. (b) error distribution on the x, y, z axes in the street06 sequence. (c) APE result on street06 sequence. (d) The trajectory of the street08 sequence. (e) error distribution on the x, y, z axes in the street08 sequence. (f) APE result on street08 sequence.

To further demonstrate the accuracy of the trajectory generated by our proposed method, we present a comparison between the estimated trajectories of our method, ground truth trajectories, and the trajectories estimated by the LOAM, F-LOAM, and BALM algorithms on the street06 and street08 sequences of the M2DGR dataset. As shown in Fig 3, which includes trajectory plots and error distribution graphs, our method exhibits superior overall trajectory consistency compared to the other algorithms, particularly in terms of the x, y, and z axes. The trajectory errors of each algorithm are shown in Table 2. BALM fails in the middle, so no result is shown. From these, we can conclude that our method achieves unbiased positioning estimation.

**Table 2:** Error comparison in the M2DGR datasets (in units of meters).

Sequence	LOAM			F-LOAM			BALM			OURS		
	MEAN	MEDIAN	RMSE	MEAN	MEDIAN	RMSE	MEAN	MEDIAN	RMSE	MEAN	MEDIAN	RMSE
Street_06	0.438	0.417	0.456	0.670	0.619	0.734	×	×	×	0.367	0.369	0.391
Street_08	3.892	3.600	4.587	28.600	27.507	31.655	×	×	×	1.529	1.317	1.787

### Dense mapping results

**Fig 4:**
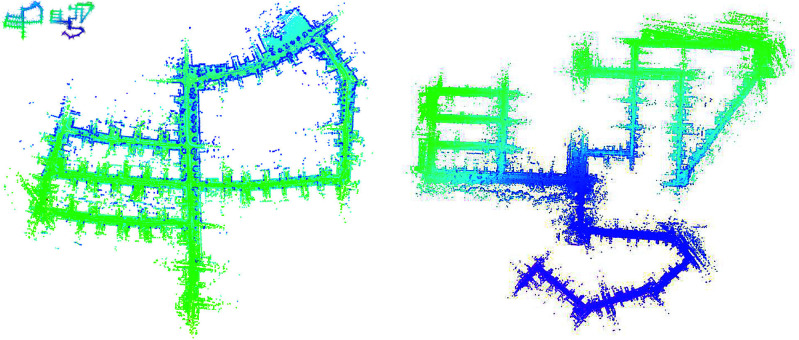
The mapping results on the dataset sequences.

To show the mapping performance of our proposed method, part of the mapping results are presented in Fig 4. We can tell that the map is locally and globally consistent. However, Point cloud maps alone cannot be directly used for subsequent robot navigation, as they lack explicit information on which areas are passable and which are not. In contrast, mesh models are crucial for robot navigation because they not only provide a more accurate surface representation of the environment but also offer essential information about traversability and non-traversability of areas. By utilizing mesh models, we can capture rich surface details, such as complex architectural structures and terrain variations, which are vital for performing collision detection and obstacle avoidance. This allows the robot to better understand its environment, ensuring safer and more efficient navigation and inspection tasks in subsequent operations.

**Fig 5:**
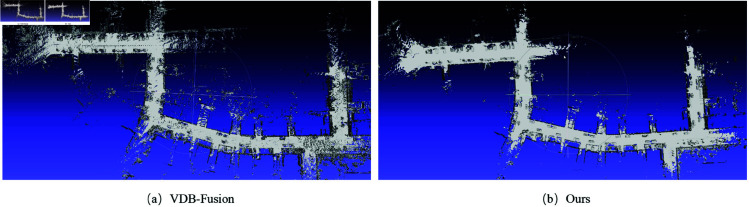
The dense mapping results on the KITTI Seq 00 (part).

Therefore, in order to further construct dense maps for inspection robot navigation, we employed the VDB-Fusion module to build high-precision 3D map models. The difference is that we directly compute SDF values by point-to-plane distance computing instead of the voxel-to-LiDAR beam end strategy in the ray-casting algorithm. The results are shown in Fig 5. Our method demonstrates significant improvements in reconstructing clear three-dimensional scenes of the real environment compared to the cluttered maps generated by VDB-Fusion. It is evident that our approach produces a more precise mesh model compared to the raw VDB-Fusion.

### ScanContext-ICP evaluation

**Fig 6:**
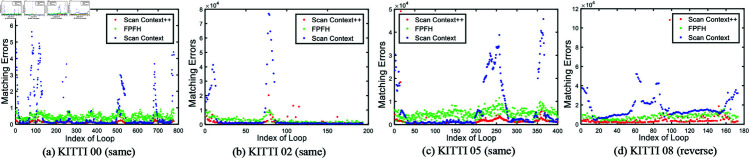
The scan matching results on the KITTI sequences.

**Fig 7:**
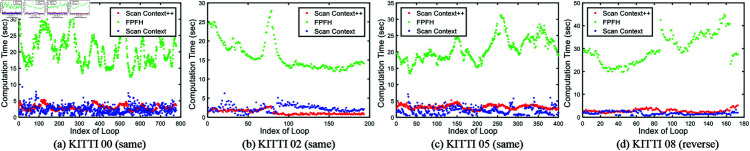
The cost time of scan matching process on the KITTI sequences.

**Fig 8:**
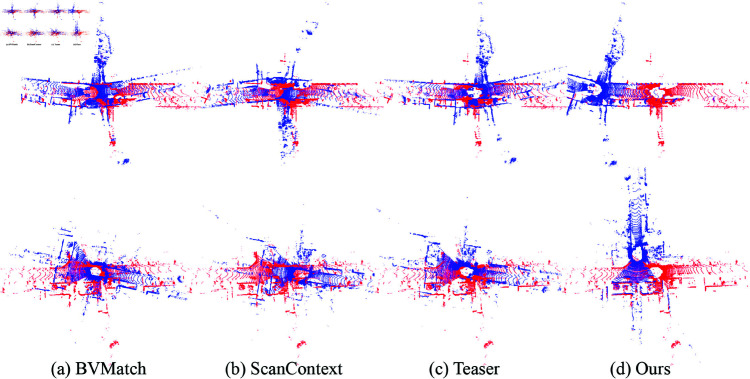
The scan matching results KITTI LiDAR scans.

To highlight the superiority of the proposed ScanContext-ICP method, we introduce a new evaluation metric for scan matching, defined as the sum of distances between points in the source and target point clouds using point-to-surfel distance. This metric allows us to assess the scan matching performance for detected true-positive looped scans. The resulting matching errors are shown in Fig 6. Among the four sequences, our method achieves fewer matching errors compared with other two methods. While ScanContext struggles to accurately match some looped scans, our ScanContext-ICP method leverages an initial alignment based on local descriptor matching followed by a refinement step using the ICP technique. This dual-step approach ensures that our method achieves better initial scan alignment and significantly reduces matching errors. The ScanContext++ (red points) consistently exhibit lower error values compared to the FPFH (green) and ScanContext (blue) points, particularly in the presence of large viewpoint changes, as demonstrated in KITTI 08 (reverse). These findings suggest that our method is more robust to variations in viewpoint, which are common in real-world scenarios. The cost time of the scan matching process is also given in Fig 7. FPFH based method is less efficient than our method. Compared with the Scancontext-based scan matching method, out method is also competitive in terms of efficiency. For global alignment, we utilize two pairs of scans for evaluation, with baselines including BVMatch [64], ScanContext, and Teaser [65]. BVMatch converts the LiDAR frame into a birdview image and extracts visual keypoints, performing frame alignment through Bag-of-words-based keypoint association. In contrast, Teaser reformulates the registration problem using a truncated least squares (TLS) cost, which makes the estimation less sensitive to spurious correspondences. It also employs a graph-theoretic framework to decouple scale, rotation, and translation, solving each transformation in sequence. Both methods represent state-of-the-art global registration techniques. Despite their strengths, neither of these methods outperform our ScanContext-ICP approach in terms of robustness and alignment accuracy. As shown in Fig 8, our method handles significant viewpoint changes more effectively. In the process of initial scan alignment, it is normal for the current viewpoint to differ from the one used when constructing the map. Our method is capable of managing these variations, achieving more accurate and stable LiDAR scan alignments than the baselines.

### Map maintenance results

**Fig 9:**
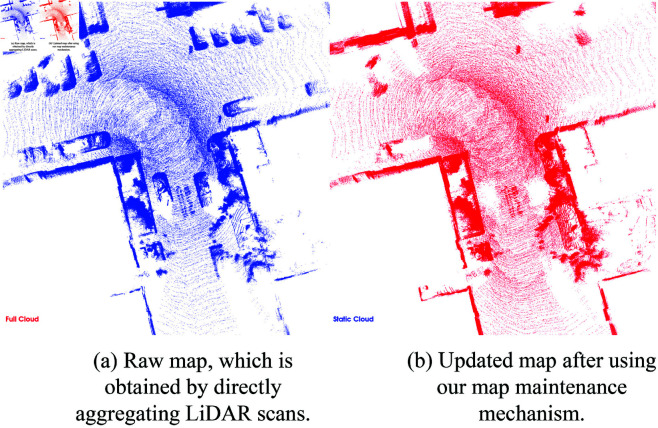
The map maintenance results in local map.

The function of map maintenance is to distinguish environment changes and update the map. In this paper, environmental changes refer mainly to dynamic elements in the testing environments, especially vehicles that appear or disappear over time. For instance, in datasets such as KITTI, parked or moving cars often introduce inconsistencies in the point cloud. When the robot revisits the same location, the absence of previously present vehicles or the presence of new ones can negatively affect position recognition and map consistency. As shown in Fig 9, our Map Maintenance Module is capable of effectively removing corresponding point cloud data while preserving the static background. This allows our map to maintain accuracy and consistency over an extended period, providing a reliable foundation for robot navigation.

## Conclusion and feature work

Navigating in the task environment and percepting the environmental information are the critical requirements for inspection robot. To achieve these functions, we propose a system capable of executing long-term localization and map maintenance. Our approach involves formulating the SLAM system within a hierarchical framework, thereby empowering it to effectively map extensive environments while curbing drift errors. By mitigating drift errors within local maps, our focus shifts towards preserving the relative transformations between these maps, eliminating the need for regenerating the global voxel map following each global trajectory correction. Furthermore, we have devised a novel Iterative Closest Point (ICP) method, integrated with the ScanContext++ descriptor. It can effectively help the ICP construct robust and accurate points’ global correspondences and thus make the ICP converge to the global optimal solution. Additionally, we introduce a time-distribution distance approach between LiDAR segments and the local map. This mechanism effectively encodes timestamp cues and models the life of each segment, providing a novel solution for map maintenance. Through extensive validation in our experiments, we demonstrate that our system fully supports inspection robots for long-term navigation in task environments.

However, the proposed method has certain limitations. While the system demonstrates strong performance, it faces several real-world challenges that must be addressed for broader deployment. First, variations in environmental conditions, such as changes in lighting and weather, can affect the quality of LiDAR data. Second, sensor inaccuracies, including noise and limited range, may influence the accuracy of the generated maps. Lastly, the algorithm was evaluated solely on a limited set of datasets, and its performance has not been fully tested across a broad range of real-world environments. Future work will involve extensive validation in diverse real-world scenarios, with a focus on enhancing system scalability, optimizing real-time performance on resource-constrained robotic platforms, and integrating complementary sensors, such as IMUs and cameras, to improve robustness in complex field conditions. Furthermore, the system’s applicability will be extended to a wider range of large-scale environments, such as power grid inspections, 3D reconstruction of urban infrastructure, and autonomous navigation in large warehouses. These applications may require further adjustments to sensor configurations and calibration methods to ensure reliable and consistent performance across diverse use cases.
